# Urethral realignment with maximal urethral length and bladder neck preservation in robot-assisted radical prostatectomy: Urinary continence recovery

**DOI:** 10.1371/journal.pone.0227744

**Published:** 2020-01-13

**Authors:** Ji Eun Heo, Jong Soo Lee, Hyeok Jun Goh, Won Sik Jang, Young Deuk Choi

**Affiliations:** 1 Department of Urology and Urological Science Institute, Yonsei University College of Medicine, Seoul, Republic of Korea; 2 Department of Urology, Dong-A University College of Medicine, Busan, Republic of Korea; University Medical Center Utrecht, NETHERLANDS

## Abstract

**Purpose:**

To evaluate early recovery of urinary continence after robot-assisted radical prostatectomy (RARP) with urethral realignment using bladder neck preservation (BNP) and maximal urethral length preservation (MULP).

**Methods:**

Patients who underwent RARP between 2014 and 2017 owing to prostate cancer with a Gleason score ≤ 7 (3+4), ≤ cT2c stage, and prostate-specific antigen level < 20 ng/ml were investigated. Patients with tumors of the bladder neck or apex on magnetic resonance imaging were excluded. A total of 266 patients underwent the operation using the standard method between 2014 and 2015 (group 1), while 305 patients underwent urethral realignment between 2016 and 2017 (group 2). Continence was defined as wearing no pad or one security pad.

**Results:**

The continence rates immediately after Foley catheter removal, at 2 weeks, and at 1, 3, 6, and 12 months after operation in group 2 were 46.9%, 63.0%, 73.4%, 90.1%, 94.8%, and 98.7%, respectively. The continence rate at 1 month in group 2 was significantly higher than that in group 1 (65.4% versus 73.4%, *p* = 0.037). The multivariate regression analysis showed that age and surgical method were factors affecting early continence recovery. The positive surgical margin rates were 18.0% and 14.8% in groups 1 and 2, respectively (p = 0.288). Biochemical recurrence occurred in 14.7% and 8.2% in groups 1 and 2, respectively (p = 0.015).

**Conclusion:**

Urethral realignment using BNP and MULP resulted in rapid continence recovery and good oncological results after RARP in young patients with a Gleason score ≤ 7 and organ-confined disease.

## Introduction

Robot-assisted radical prostatectomy (RARP) is one of the standard surgical treatments for clinical localized prostate cancer (PCa). Optimal cancer control is the primary goal of RARP; nevertheless, RARP also aims to preserve urinary continence. Robotic technology enables surgeons to execute precise movements for the preservation of anatomical structures essential for urinary continence and potency. In a previous systematic review and meta-analysis of urinary continence after RARP, the 12-month urinary incontinence rates ranged from 8% to 11% (mean, 9%) among the included studies that defined continence as wearing no pad or safety pad [1].

Urinary incontinence may have a serious negative effect on the quality of life among patients undergoing radical prostatectomy (RP) [1]. Although the complicated physiology of mechanisms related to urinary continence after RP remains unclear, bladder neck preservation (BNP) is considered to play the most important role [2–4]. However, the extent of BNP should be considered because aggressive BNP could be associated with higher positive surgical margin (PSM) rate, particularly for non-organ-confined cancers [5, 6]. With respect to both optimal cancer control and urinary continence, maximal BNP is a reasonable surgical technique for patients with clinically organ-confined cancer and Gleason score (GS) ≤ 7 (3+4).

Maximal urethral length preservation (MULP) has been shown to ensure early return of continence [7–9]. Increased urethral length, which includes a greater number of smooth muscle fibers and rhabdosphincters, aids in the functional recovery of the rhabdosphincter [10]. Thus, a combination of BNP and MULP techniques to attain urethral realignment may maximize the functional urethral length and achieve early recovery of urinary continence.

The present study aimed to evaluate recovery of urinary continence after RARP with urethral realignment using BNP and MULP techniques compared with that using the standard technique of RARP. The primary endpoint was to assess the urinary continence rate at different time points, whereas the secondary endpoint was to examine oncological outcomes.

## Materials and methods

This study was approved by the institutional review board (IRB) of Yonsei University Severance Hospital (IRB number: 4-2019-0106) and the requirement for informed consent was waived. The patient records and information were anonymized prior to analysis. Data were retrospectively collected from patients who underwent RARP for PCa at our institution from January 2014 to December 2017. Multiparametric magnetic resonance imaging (MRI) of the prostate was performed in all cases. Patients with localized PCa (clinical stages T1–T2cN0M0), GS ≤ 7 (3+4), and prostate-specific antigen (PSA) level < 20 ng/ml were investigated [11]. For patients whose tumors were suspected to be located on the bladder neck or apex on MRI, the urethral realignment technique was not performed. The standard technique of RARP was performed in cases treated between 2014 and 2015 (group 1; n = 266), that patients with tumors of the bladder neck or apex on MRI also excluded. Patients treated between 2016 and 2017 underwent the surgeries with urethral realignment technique using BNP and MULP techniques (group 2; n = 305). A single surgeon (Y.D.C.) with experience of performing more than 3,000 RARPs performed all surgeries. Planned procedures were discussed with each patient, from whom informed consent was obtained. Preoperative functional parameters were assessed using the International Prostate Symptom Score questionnaires.

The technique of BNP in RARP has been previously described [12]. In brief, the anterior bladder is tented by traction of the cephalad part of the detrusor muscle to form a ridge at the detrusor apron. Using a combination of sharp and blunt dissection to tease bladder muscle fibers away from the prostate, a funneling bladder neck is created. After dissecting anteriorly and circumferentially, the anterior side of the bladder neck are incised as distally as possible.

The procedure of MULP in RARP was described by Hamada et al [10]. In brief, after dissecting the dorsal vein complex, the apex and the rhabdosphincter are seen. Toward the membranous urethra, the striated and smooth muscle fibers are carefully divided. By releasing the fibrous connections of the prostate at the apex, an additional length of the intra-abdominal urethra is obtained.

**Fig 1:**
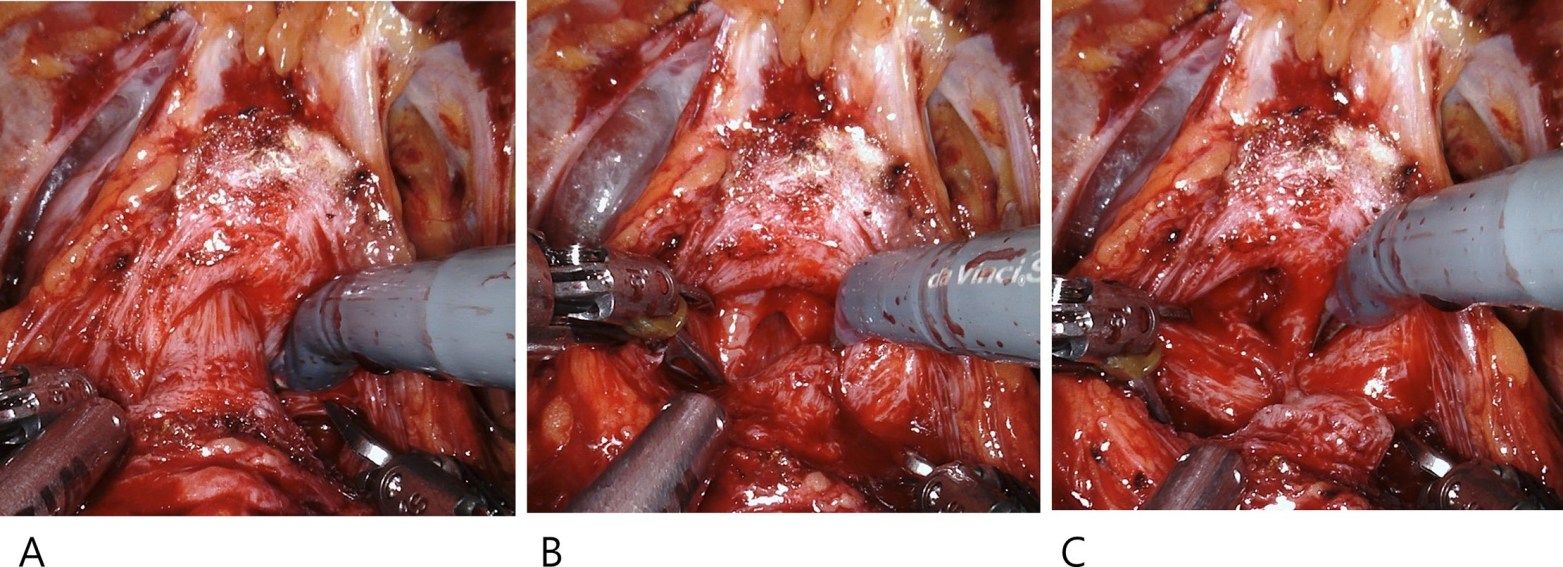
Bladder neck preservation and proximal urethral isolation.

Our surgeries were performed using the extraperitoneal approach [13]. The endopelvic fascia was minimally dissected. The BNP technique employed was similar to that previously described [12]. With incision of the detrusor muscle fibers at the insertion on the ventral surface of the prostate base, athermal dissection was continued until the longitudinal smooth muscle component of the urethra was identified. Subsequently, the bladder neck was pulled cephalad, and the proximal urethra was isolated. The isolated urethra was incised just below the verumontanum. After the dorsal side of the prostate base was dissected from the bladder neck using cold scissors, the bladder neck and proximal urethra were completely preserved, and the intraoperative urethral length measured as approximately 10 mm (Fig 1).

**Fig 2:**
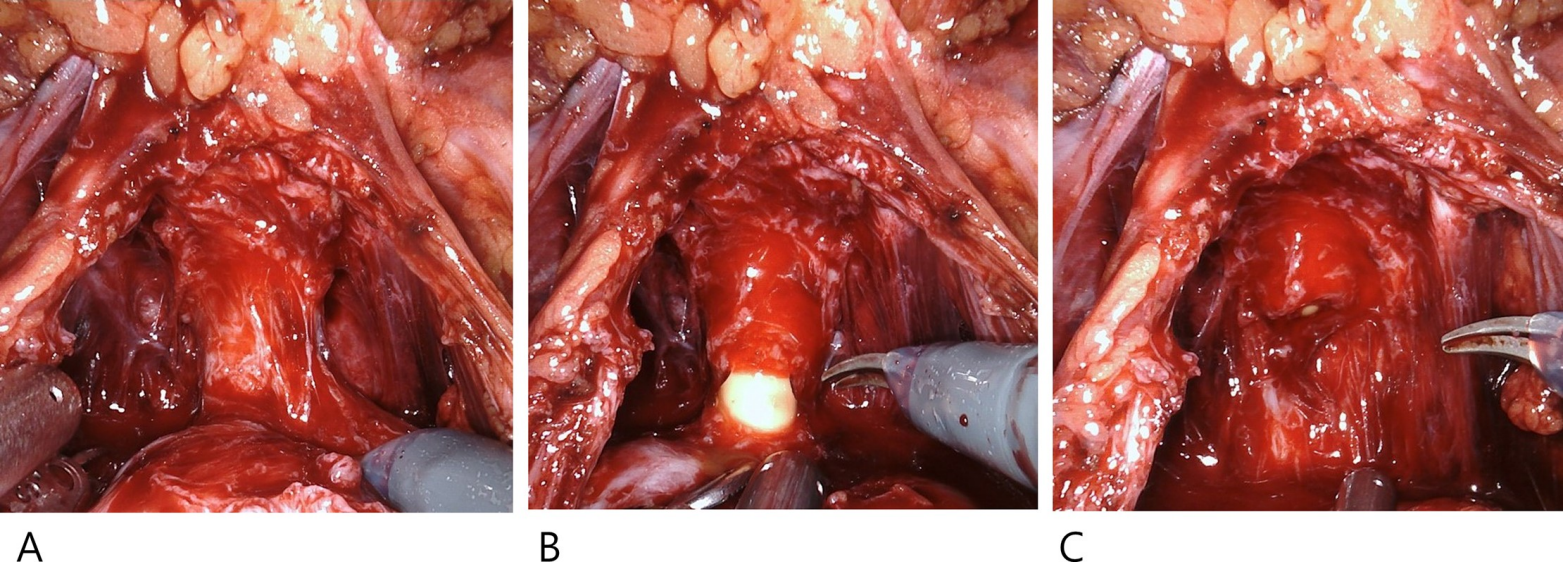
Preservation of maximal distal urethral length and pelvic floor tissue.

The seminal vesicles were dissected from Denonvilliers’ fascia. The dorsal surface of the prostate was bluntly dissected toward the apex, with the seminal vesicles being pulled. The detrusor apron that covered the anterior surface of the prostate was incised, whereas the puboprostatic ligaments were preserved, and the dorsal vein was not ligated. The prostate was pulled cephalad, and the prostatic apex was released from the fibrous and muscular connective tissues around the urethra [10]. The distal urethra was maximally isolated and incised just below the prostatic apex using cold scissors. The lateral surface of the prostate was dissected with minimal electrocauterization, and the pelvic floor tissue was preserved. (Fig 2).

Neurovascular bundle preservation was routinely accomplished, and pelvic lymph node dissection was not performed in this cohort.

**Fig 3:**
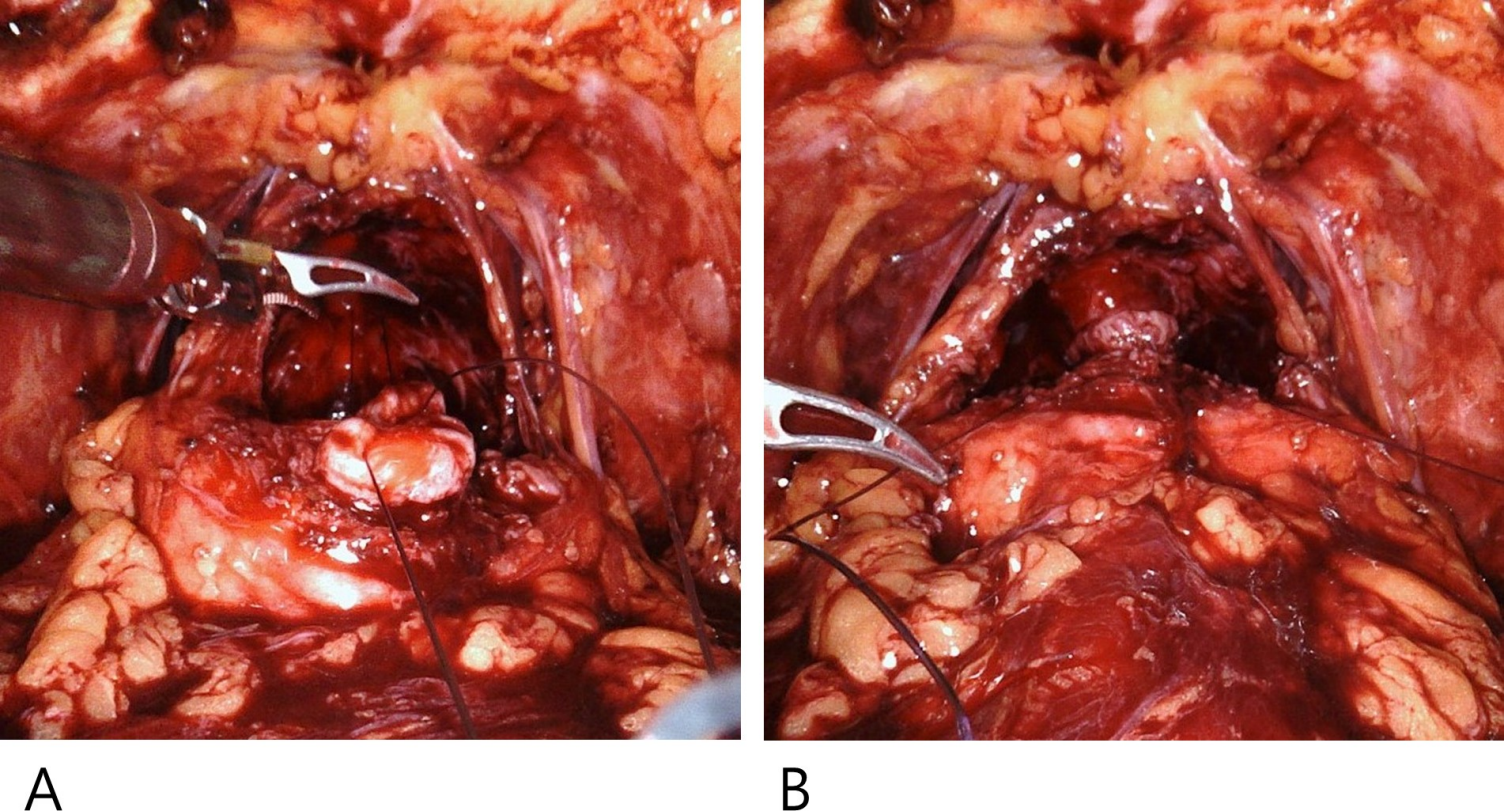
Urethral realignment.

The proximal and distal urethra was anastomosed using continuous 3–0 monofilament suture (Fig 3).

**Fig 4:**
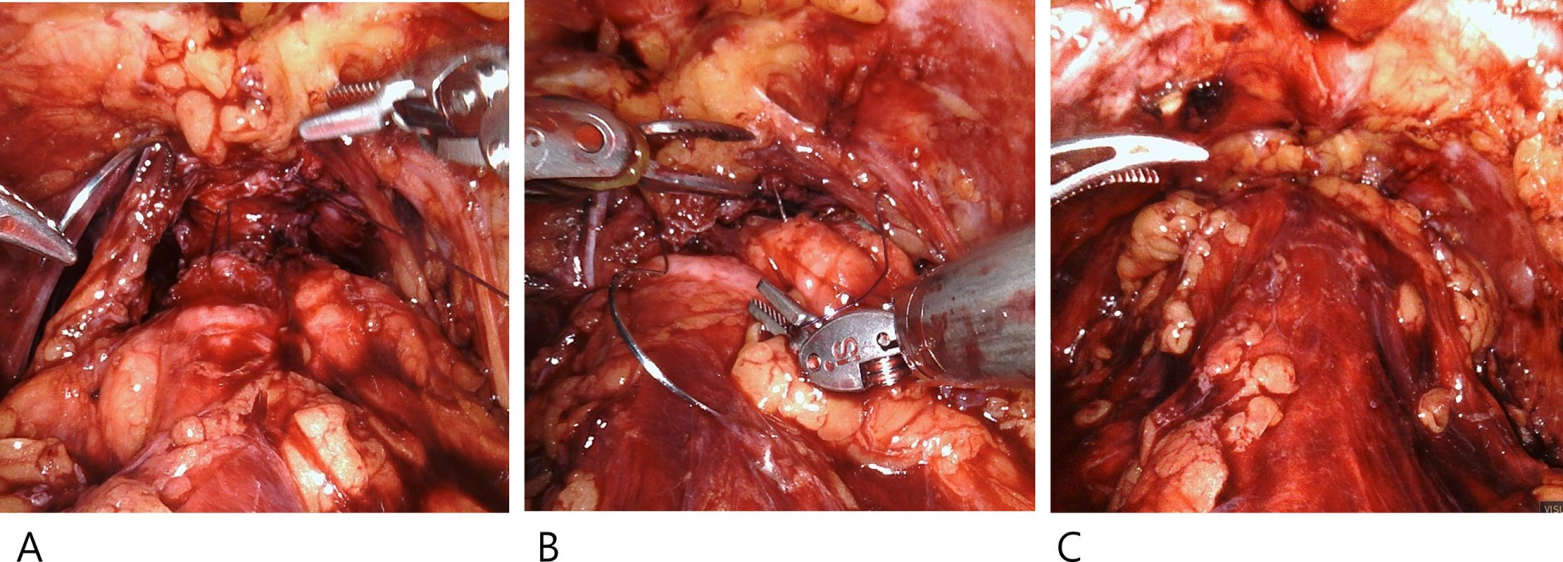
Anterior reconstruction.

After urethral realignment, anterior reconstruction was completed by reattaching the puboprostatic ligaments and the deep dorsal vein complex to the anterior distal bladder [14]. The dissected space around the bladder was closed by suturing the anterior bladder to the arcus tendineus. Anterior reconstruction was also performed using the standard technique. Anastomotic integrity was routinely checked by distending the bladder using 150 ml of saline to confirm the absence of visible leakage (Fig 4).

The extraperitoneal drainage was removed. The patients were discharged on the 2^nd^ postoperative day, whereas the catheter was removed on the 10^th^ postoperative day. All patients were instructed to perform pelvic floor muscle exercises, which were initiated from the time of catheter removal until continence recovery. The continence rate was regularly assessed by patient reporting immediately after Foley catheter removal, at 2 weeks, and at 1, 3, 6, and 12 months. Continence was defined as wearing no pad or one pad for security or occasional stress incontinence that patients self-reported. Uroflowmetry was performed at the 2-month follow-up to evaluate voiding patterns, and MRI was simultaneously performed.

Biochemical recurrence (BCR) was defined as detectable PSA after RP or any two consecutive increases of ≥ 0.2 ng/ml in PSA level with undetectable PSA after RP [15].

Continuous variables are expressed as medians (interquartile ranges), whereas categorical variables are reported as number of occurrences and frequency. Student’s *t*-test was used to compare continuous variables, whereas chi-square test was used to compare categorical variables. Parameters were estimated in univariate and multivariate logistic regression analyses to identify predictors of early continence recovery. Statistical analysis was performed using SPSS version 23.0 (IBM Corp., Armonk, NY, USA). A p-value (p) < 0.05 was considered to indicate statistical significance.

## Results

A total of 571 patients were included in this study. The patient characteristics are summarized in Table 1. There was a significant difference between the ages of the patients in the groups, with median values of 65 years and 66 years for groups 1 and 2, respectively (p = 0.014). The median prostate volume measured by transrectal ultrasonography was 30 g in group 1 and 31 g in group 2 (p = 0.165). Patients with a biopsy GS 6 were 66.5% and 58.4% for groups 1 and 2, respectively (p = 0.047). No significant differences in body mass index, PSA level, clinical stage, and baseline urinary function were observed between the two groups (p = 0.120, 0.165, 0.375 and 0.928, respectively).

**Table 1:** Patients’ characteristics.

	Group 1^a^	Group 2^a^	p-value
No. of patients	266	305	
Age, years (median [IQR])	65 (59–69)	66 (61–71)	0.014
Body mass index, kg/m^2^ (median [IQR])	24.16 (22.15–25.47)	24.22 (23.18–25.82)	0.120
Prostate volume, g (median [IQR])	30 (25–40)	31 (26–42)	0.165
PSA, ng/ml (median [IQR])	6.67 (4.79–10.20)	6.47 (4.86–9.92)	0.893
Biopsy Gleason score			0.047
6	177 (66.5)	178 (58.4)	
7 (3+4)	89 (33.5)	127 (41.6)	
Clinical stage			0.375
T1c	63 (23.7)	55 (18.0)	
T2a	77 (28.9)	94 (30.8)	
T2b	57 (21.4)	76 (24.9)	
T2c	69 (25.9)	80 (26.2)	
IPSS (median [IQR])	15 (9–20)	14 (9–19)	0.928

^a^ Group 1: Standard method, Group 2: Urethral realignment method

Data are expressed as N (%) unless otherwise specified.

IPSS, International Prostate Symptom Score; IQR, interquartile range; PSA, prostate-specific antigen.

The median operating times were 101 min and 97 min in groups 1 and 2, respectively (p = 0.302). The median console times were 34 min in group 1 and 32 min in group 2 (p = 0.007). The median value of estimated blood loss was 300 cc in both groups (Table 2).

**Table 2:** Intraoperative variables, pathological data, and biochemical recurrence.

	Group 1^a^	Group 2^a^	p-value
Operating time, min (median [IQR])	101 (84–120)	97 (88–115)	0.302
Console time, min (median [IQR])	34 (28–40)	32 (29–38)	0.007
EBL, ml (median [IQR])	300 (200–500)	300 (150–500)	0.049
Pathological Gleason			0.086
6	93 (35.0)	80 (26.2)	
7 (3+4)	122 (45.9)	157 (51.5)	
7 (4+3)	29 (10.9)	46 (15.1)	
8–10	22 (8.3)	22 (7.2)	
Pathological stage			< 0.001
T2	198 (74.4)	263 (86.2)	
T3–4	68 (25.6)	42 (13.8)	
PSM	48 (18.0)	45 (14.8)	0.288
Apex	32 (12.0)	23 (7.5)	0.070
Base	18 (6.8)	22 (7.2)	0.835
Biochemical recurrence	39 (14.7)	25 (8.2)	0.015

^a^ Group 1: Standard method, Group 2: Urethral realignment method

Data are expressed as N (%) unless otherwise specified.

EBL, estimated blood loss; IQR, interquartile range; PSM, positive surgical margin.

The pathological results are presented in Table 2. The rates of pathological GS ≥ 7 (4+3) were not different between the two groups (19.2% vs. 22.3%, p = 0.086). The rates of pathological stage ≥ T3a were 25.6% in group 1 and 13.8% in group 2 (p < 0.001). The overall PSM rates were 18.0% and 14.8% in groups 1 and 2, respectively. The PSM rates according to location in group 2 were 7.5% at the apex and 7.2% at the base, with the PSM rates not significantly different from those in group 1 (apex; p = 0.070, base; p = 0.835). BCR occurred in 39 (14.7%) of patients from group 1 and in 25 (8.2%) from group 2. The difference in BCR rates between both groups was statistically significant (p = 0.015). Local recurrence was not detected in the follow-up image study.

The continence rates at immediate follow-up were 38.3% and 46.9% for groups 1 and 2, respectively (p = 0.040). The continence rates gradually improved at 2 weeks, and 1, 3, 6, and 12 months in both groups (group 1: 55.6%, 65.4%, 92.5%, 95.8%, 97.7%; group 2: 63.0%, 73.4%, 90.1%, 94.8%, 98.7%, respectively). The continence rates at 1 month were significantly higher in group 2 than in group 1 (p = 0.037). According to the previously described definition of urinary continence, 6 patients in group 1 and 4 in group 2 still used more than one pad per day at the 12-month follow-up (Table 3). One patient in group 1 underwent insertion of an artificial urethral sphincter.

**Table 3:** Continence rates and uroflowmetry results.

	Group 1^a^	Group 2^a^	p-value
**Continence rate at different time points**			
Immediate	102 (38.3)	143 (46.9)	0.040
2 weeks	148 (55.6)	192 (63.0)	0.076
1 month	174 (65.4)	224 (73.4)	0.037
3 months	246 (92.5)	273 (90.1)	0.218
6 months	255 (95.8)	289 (94.8)	0.533
12 months	260 (97.7)	301 (98.7)	0.391
Not pad-free	6 (2.3)	4 (1.3)	
**Uroflowmetry results at 2 months**			
Q_max_, ml/s (median [IQR])	12.3 (8.3–18.3)	14.8 (9–22.3)	0.037
VV, ml (median [IQR])	179 (122–276)	195 (115–294)	0.531
PVR, ml (median [IQR])	0 (0–10)	0 (0–0)	0.001

^a^ Group 1: Standard method, Group 2: Urethral realignment method

Data are expressed as N (%) unless otherwise specified.

PVR, post-void residual volume; Q_max_, maximum urinary flow rate; VV, voided volume.

Uroflowmetry results are shown in Table 3. The median maximum urinary flow rate (Q_max_) was 12.3 ml/s in group 1 and 14.8 ml/s in group 2. The median voided volume was 179 ml in group 1 and 195 ml in group 2. The median post-void residual volume was 0 ml in both groups.

Univariate regression analysis revealed that age (hazard ratio [HR] = 0.922 [95% confidence interval 0.897–0.949], p < 0.001) and surgical method (HR = 1.462 [1.022–2.092], p = 0.038) were predictors of continence at 1 month after surgery. Both variables were significant predictors in multivariate regression analysis (age: HR = 0.917 [0.891–0.943], p < 0.001; surgical method: HR = 1.740 [1.193–2.536], p = 0.004; Table 4).

**Table 4:** Univariate and multivariate regression analysis of continence at 1 month.

Variables		Univariate			Multivariate	
	HR	95% CI	p-value	HR	95% CI	p-value
Age	0.922	(0.897–0.949)	< 0.001	0.917	(0.891–0.943)	< 0.001
Body mass index	1.016	(0.948–1.088)	0.661			
Prostate volume	0.994	(0.983–1.006)	0.330			
PSA	1.001	(0.988–1.014)	0.890			
Clinical stage						
T1c	1	(reference)				
T2a	1.438	0.848–2.438	0.178			
T2b	0.753	0.444–1.275	0.290			
T2c	0.924	0.549–1.556	0.766			
IPSS	0.995	(0.971–1.019)	0.687			
Surgical method						
Standard	1	(reference)		1	(reference)	
Urethral realignment	1.462	(1.022–2.092)	0.038	1.740	(1.193–2.536)	0.004
Operating time	0.997	(0.991–1.004)	0.415			
Console time	0.984	(0.965–1.004)	0.118			
EBL	1.000	(0.999–1.001)	0.688			
Pathologic stage						
T2	1	(reference)				
T3-4	0.675	(0.437–1.044)	0.078			

CI, confidence interval; EBL, estimated blood loss; HR, hazard ratio; IPSS, International Prostate Symptom Score; PSA, prostate-specific antigen.

## Discussion

Considering the negative effect of urinary incontinence after RP on patients’ quality of life, urinary continence recovery after RP is increasingly considered to be as important as cancer control [16]. Accurate robot-assisted surgery is available to minimize postoperative complications and preserve the anatomical structures associated with urinary incontinence. Thus, RARP can achieve improved results in continence recovery compared to the open or laparoscopic approach [1]. The complex mechanism of recovery from urethral symptoms after RP is not yet fully understood; nonetheless, it is widely accepted that surgeons should preserve the bladder neck, rhabdosphincter, and periurethral supporting structures, and perform nerve-sparing surgery and reconstruction. Several surgical procedures have been proposed for this purpose [9, 10, 14, 17–21].

Various mechanisms have been considered responsible for continence after RP, and BNP appears to play the most important role [2–4]. The significance of bladder neck for continence was shown in a study on cases of traumatic posterior urethral injury [22]. MULP can preserve the rhabdosphincter, which is located between the verumontanum and the distal edge of the prostatic apex. Hamada et al. [10] reported a continence rate of 70% for MULP at 1 month after surgery. By leaving the urethral stump longer, vesicourethral anastomosis can be facilitated, and bladder descent can be reduced. In our study, this advantage can be further maximized through BNP, and urethral realignment can be achieved using combined BNP and MULP techniques. Moreover, anterior reconstruction provides anatomical support for the stabilization of the urethra and rhabdosphincter in their anatomical position [17]. Hence, we performed anterior reconstruction for all the patients.

In a systematic review and meta-analysis of continence recovery after RARP, the 12-month continence rates ranged from 89% to 92% among studies that used “no pad or safety pad” as the definition of continence. In addition, studies involving approximately 100 cases reported 1-month continence rates of 33–86% [1]. In our study, continence rates at immediate follow-up and at 1 month in group 2 were significantly higher than those in group 1. Continence rates at 12 months showed no significant difference between the two groups; nonetheless the continence rates in group 2 were higher than those in group 1. Thus, continence rates within 1 month during the early period improved in patients treated using the urethral realignment technique.

Notably, this technique is not always feasible because of the higher risk of PSM. Patients with tumors located on the bladder neck or apex on MRI or with suspected extraprostatic extension should be excluded. Preoperative selection of patients using MRI ensures that the overall PSM rate is comparable to that in previously published results [13, 21]. In addition, the PSM and BCR rates in group 2 were not different from those in group 1, and local recurrence was not observed. Furthermore, despite the significant difference in the pathological stage of the two group, clinical and pathological stages were not significant predictors for early continence recovery. Thus, the urethral realignment technique showed satisfactory oncological outcomes compared with that in the standard technique.

One urethral stricture was recorded in the urethral realignment method; albeit, this complication was successfully resolved after two sessions of urethral dilation. Considering the total number of patients included in the study, urethral realignment and reconstruction of the periurethral structures ensured a water-tight anastomosis without increasing the risk for strictures in almost all cases.

The present study has several limitations. First, the sample selection was not randomized, and the majority of patients are classified as low risk [11]. Available treatment plans were discussed with the patients, and men who decided to undergo the operations were investigated. Second, the prostate volume was small; however, these volumes were comparable to those in previous studies on Korean men [23, 24]. Finally, the presence of comorbidities that could potentially affect continence status was not recorded in our database. Notwithstanding these study limitations, our study showed promising results with respect to early continence recovery in patients after RARP using urethral realignment with combined BNP and MULP techniques.

## Conclusion

Urethral realignment with combined BNP and MULP techniques resulted in rapid continence recovery after RARP compared to the standard method. These results were possible owing to the preservation of the anatomical and full functional lengths of both internal and external urinary sphincters. The use of our technique in young patients with GS ≤ 7 and organ-confined PCa led to early recovery and produced good oncological results. However, high-risk patients or those with suspected tumors located on the bladder neck or apex on MRI should not be treated using this technique.
